# Recent advances of DNA methylation in osteonecrosis of the femoral head

**DOI:** 10.3389/fgene.2025.1526852

**Published:** 2025-10-02

**Authors:** Tingting Zhou, Huan Liu, Huayan Li, Yan Zhang, Xiaoming Li

**Affiliations:** ^1^ Department of Orthopedics, Cangzhou Hospital of Integrated Traditional Chinese Medicine-Western Medicine Hebei, Cangzhou, Hebei, China; ^2^ Hebei Key Laboratory of Integrated Traditional and Western Medicine in Osteoarthrosis Research, Cangzhou, China; ^3^ Graduate School, Hebei University of Chinese Medicine, Shijiazhuang, China; ^4^ Spine Disease Research Institute, Longhua Hospital, Shanghai University of Traditional Chinese Medicine, Shanghai, China; ^5^ Key Laboratory of Theory and Therapy of Muscles and Bones, Ministry of Education, Shanghai, China

**Keywords:** osteonecrosis of the femoral head, DNA methylation, osteoblast differentiation, angiogenesis, osteocyte apoptosis

## Abstract

DNA methylation, maintenance, and demethylation are essential for maintaining normal physiological functions. Recent studies have revealed that DNA methylation plays a crucial role in the progression of osteonecrosis of the femoral head. DNA methylation regulates the differentiation direction of bone marrow mesenchymal stem cells, affects angiogenesis, and is involved in the proliferation and apoptosis of osteocytes, holding significant potential for early diagnosis and treatment of the disease. This paper introduces the concept and process of DNA methylation, with an emphasis on its molecular mechanisms in osteonecrosis of the femoral head. Furthermore, we propose that modulating different states of DNA methylation, such as inhibiting the function of DNA methyltransferases to induce DNA demethylation, could impact the disease progression of osteonecrosis of the femoral head, offering new insights for its treatment.

## 1 Introduction

Osteonecrosis of the femoral head (ONFH) is a debilitating condition influenced by both environmental and genetic factors, characterized by a prolonged disease course, a low cure rate, and significant disability. The global incidence of ONFH is increasing. Approximately 20,000 new cases are annually diagnosed in the United States, with a cumulative prevalence ranging from 300,000 to 600,000 cases. The number of patients suffering from ONFH is as high as 8.12 million in China. It primarily affects young adults from 20 to 40 years old, and ranks among the most prevalent diseases leading to disability in young and middle-aged individuals (Zhao et al., 2020; George and Lane, 2022). Its main pathological process involves the interruption of blood supply to the femoral head, leading to bone death, trabecular necrosis, separation of the subchondral bone from the articular cartilage, and irreversible collapse of the femoral head, eventually resulting in pain, limited mobility, and dysfunction of the hip joint (Guerado and Caso, 2016; Chang et al., 2020).

Nonoperative treatment modalities have limited effectiveness in halting the progression of ONFH and are only applicable to a small number of patients with small-sized and medial lesions (Mont et al., 2010; Mont et al., 2020). Recent studies have evaluated the efficacy of pharmacological therapy and biophysical treatments. Statins may potentially reduce the risk of ONFH, by modulating lipid metabolism and the expression of genes related to bone differentiation (Pritchett, 2001; Ajmal et al., 2009; Ren et al., 2020; Wu et al., 2024). Meanwhile, electrical stimulation showed beneficial effects in managing early-stage ONFH, and improving clinical and radiographic parameters (Bradley et al., 1986; Fornell et al., 2018; Yamamoto et al., 2018; Ellenrieder et al., 2023). However, considering that these data originate from small-scale and single-center studies and only provide low-level evidence, the results in these nonoperative studies are inconclusive. In contrast, joint-preserving procedures, such as core decompression, adjunctive bone-grafting, small-diameter drilling, and vascular pedicle bone transplantation, should be attempted to save the femoral head and delay progression in early-stage lesions (Microsurgery Department of the Orthopedics Branch of the Chinese Medical Doctor Association et al., 2017). Additionally, core decompression (CD) procedures have been applied for more than 50 years, and are more effective than non-surgical treatments. With an overall success rate of approximately 65% (Hua et al., 2019), CD is widely recommended for patients with a lesion area of less than 30% of the femoral head volume (Roth et al., 2016). In recent years, cell-based adjuvant therapy (such as autologous bone marrow aspiration concentrate) has further improved the clinical efficacy of CD (Piuzzi et al., 2017; Wang et al., 2023). However, the standardization of cell-based therapy still faces challenges, including heterogeneity in cell collection, processing, and transplantation methods, as well as differences in cell quality among patients. When the disease progresses to the advanced stage, patients often require total hip arthroplasty (Mont et al., 2020; Zhao et al., 2020). However, for young patients, the most optimal therapeutic objective is to maintain the structural integrity of the femoral head. The effective treatment of ONFH at the early stage remains a highly challenging. At present, consensus is lacking among the diverse treatment modalities. Therefore, further clarification of the pathological molecular mechanisms involved in ONFH is essential for managing this disease.

The occurrence and development of ONFH is influenced by a combination of environmental, genetic and social factors. In theory, its pathogenesis involves a multifactorial interplay of various mechanisms, including the abnormal differentiation of bone marrow-derived mesenchymal stem cells (BMSCs), blood supply disorders, lipid metabolism disorders, apoptosis and inflammation, and genetic polymorphism (Li, 2014). Nevertheless, the precise molecular mechanism has not been fully elucidated (Wang A. et al., 2018). Recent advances have shown that epigenetics, particularly DNA methylation, plays an important role in the pathological mechanism of ONFH.

Epigenetics refers to the heritable changes in chromatin states that occur without alterations in the underlying DNA sequence (Cavalli and Heard, 2019). As a fundamental epigenetic modification, DNA methylation is the most common and well-studied one at present. Disruption of the gene expression patterns mediated by this mechanism induces various diseases, such as diabetes (Bansal and Pinney, 2017), kidney disease (Reddy and Natarajan, 2015), cardiovascular diseases, bone and joint diseases, and even tumors (Koch et al., 2018; Agha et al., 2019; van Meurs et al., 2019). A comprehensive genome-wide DNA methylation analysis of human pancreatic islets revealed intriguing insights. In patients with type-2 diabetes (T2D), 276 methylated cytosine-phosphate-guanine (CpG) sites exhibited differential methylation compared with non-diabetic individuals. Notably, approximately 96% of these sites were hypomethylated (Volkmar et al., 2012). In diabetic kidney disease (DKD), the up-regulation of DNA methyltransferase 1 (DNMT1) occurred in immune cells, inducing abnormal cytosine methylation of the upstream regulators of the mammalian target of rapamycin (mTOR) pathway. Consequently, this signaling pathway became pathogenically activated, contributing to inflammation in DKD (Chen et al., 2019). Hypomethylation modification was found to be associated with poor prognosis in breast cancer. In particular, stemness and proliferation-related transcription factors, namely, OCT4, NANOG, SOX2, and SIN3, were elevated in circulating tumor cluster (CTC) cells, suggesting the effect of DNA methylation levels on tumor cell proliferation and metastasis (Gkountela et al., 2019). Moreover, DNA methylation, as a potential molecular mechanism, can affect bone remodeling and angiogenesis and participate in the occurrence and development of bone tissue diseases, including osteonecrosis of the femoral head (ONFH) (Sharma et al., 2024). For example, the active demethylation of the promoter regions of the Runx2, osteocalcin, and osterix genes, mediated by mechanisms dependent on the growth arrest and DNA-damage-inducible protein (GADD45), is intricately involved in the osteogenic differentiation of adipose-derived mesenchymal stem cells (MSCs) (Zhang et al., 2011). Man et al. exploited the impact of inducing hypomethylation through the DNA methyltransferase inhibitor 5-azacytidine (AZT), and found that it significantly increased osteogenic differentiation and mineralization of human BMSCs (hBMSCs) and enhanced the pro-angiogenic cytokine release of human umbilical vein endothelial cells (HUVECs) (Man et al., 2023).

**FIGURE 1:**
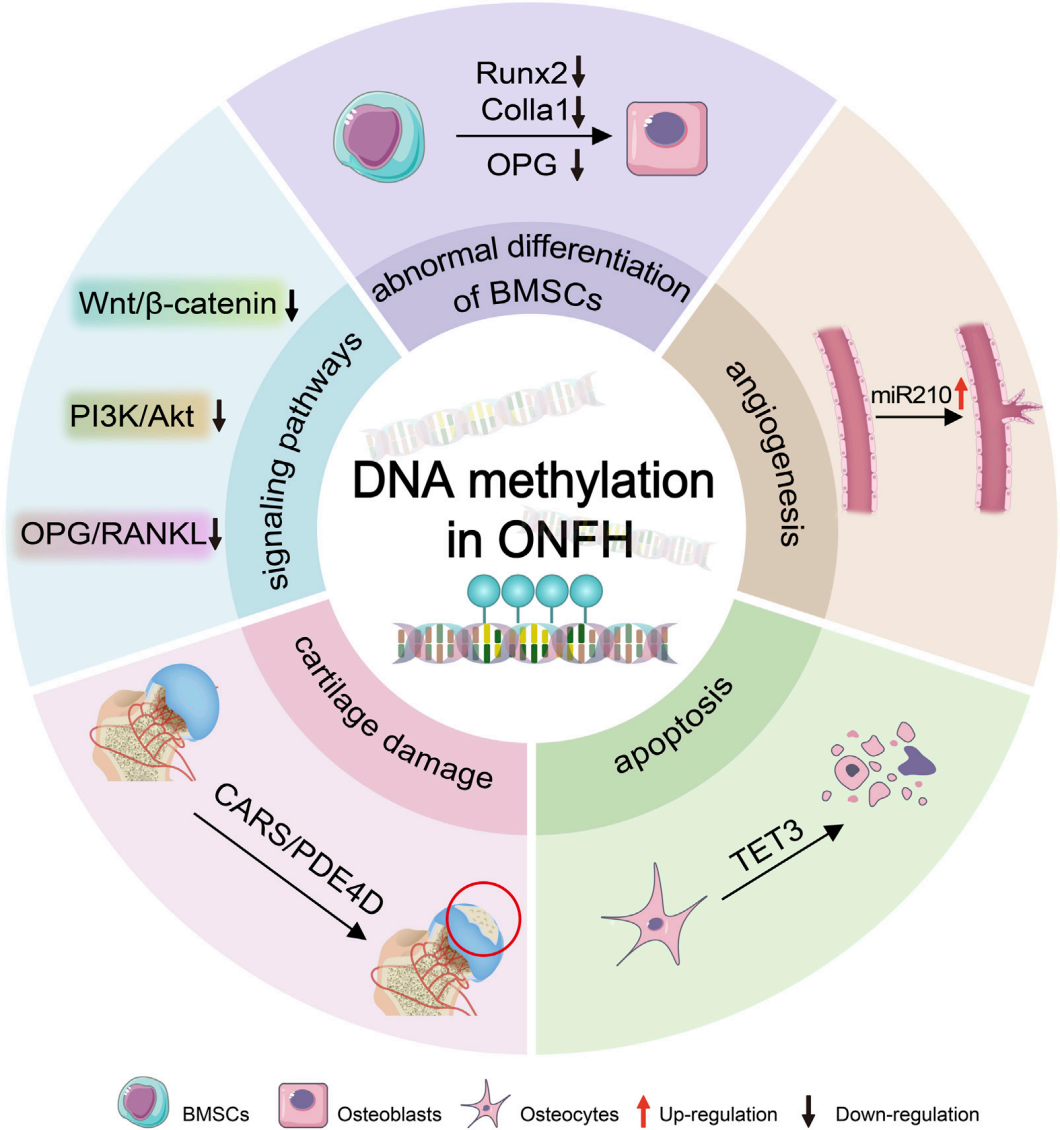
DNA methylation leads to ONFH. DNA methylation induces the progression of ONFH by epigenetically regulating osteogenesis-related genes (e.g., Runx2, Colla1, and OPG), signaling pathways (e.g., Wnt/β-catenin, PI3K/Akt, and OPG/RANKL), and miRNAs (such as miR210), thereby driving deteriorations in osteogenic differentiation of BMSCs mediated by osteogenic-related genes, impaired angiogenesis regulated by miR210, osteocytes apoptosis mediated by TET3, and cartilage degradation involved by CARS. BMSCs:bone marrow mesenchymal stem cells; ONFH: osteonecrosis of the femoral head.

We review the research on DNA methylation in ONFH to uncover its significance and potential as both a diagnostic tool and therapeutic target (Figure 1).

## 2 DNA methylation process

DNA methylation is catalyzed by a family of DNA methyltransferases (Dnmts) that transfer a methyl group from S-adenyl methionine (SAM) to the fifth carbon of a cytosine residue to form 5-methylcytosine (5mC) (Liu et al., 2018; Greenberg and Bourc’his, 2019). In the human genome, Dnmts primarily include Dnmt1, Dnmt2, Dnmt3a, Dnmt3b, and Dnmt3L. Among these, Dnmt1 and Dnmt3a/b are canonical DNA methyltransferases whereas Dnmt2 and Dnmt3L are considered non-canonical ones (Lyko, 2018). In plants, conserved Dnmts are present known as chromomethylases (CMTs). Additionally, Dnmt4, Dnmt5, and Dnmt6 have also been identified in algae and fungi (Law and Jacobsen, 2010). Human DNA methyltransferases can be categorized into two main groups. The first category includes DNMT3a and DNMT3b, which are also known as *de novo* methyltransferases. Their primary function is to methylate previously unmethylated sites, introducing methyl groups into naked DNA. The second category includes DNMT1, which acts as a DNA maintenance methyltransferase. DNMT1 is located at the replication fork during cell replication and methylates hemimethylated DNA sites to preserve the original methylation pattern before replication occurs (Moore et al., 2013). The role of Dnmt2 remains controversial. Notably, Dnmt2 can methylate tRNA molecules through a catalytic mechanism similar to DNA methylation. Conversely, Dnmt3L shares a protein structure with Dnmt3 but lacks key catalytic motifs. Instead, it functions as a cofactor for DNA methylation.

DNA methylation is a dynamic process that includes both the establishment and maintenance of DNA methylation patterns, as well as active demethylation. Dnmts add methyl groups to cytosine residues, wheras demethylation can occur through various mechanisms (Edwards et al., 2017) and is categorized into two forms: active and passive. Passive demethylation predominantly occurs in dividing cells. During cell replication, Dnmt1 maintains DNA methylation. In particular, the inhibition and/or dysfunction of Dnmt1 keeps newly incorporated cytosines unmethylated. Consequently, the overall methylation levels decrease after cell division, enabling passive demethylation.

Active DNA demethylation can occur in both dividing and non-dividing cells through chemical modifications of 5mC by DNA demethylases (deamination or oxidation) and through the base excision repair (BER) pathway. Activation-induced cytidine deaminase/apolipoprotein B mRNA-editing enzyme complex (AID/APOBEC) effectively transforms 5mC into thymine after deamination. Subsequently, the modified base is replaced with naked cytosine through the BER pathway. Additionally, proteins of the ten-eleven translocation (TET) family catalyze the conversion of 5mC to 5-hydroxymethylcytosine (5hmC), which ultimately contributes to BER-mediated demethylation (Wu and Zhang, 2017). The dynamic balance of DNA methylation and demethylation is essential for maintaining normal cellular functions.

## 3 The regulatory role of DNA methylation in osteonecrosis of femoral head

### 3.1 The impact of DNA methylation on the abnormal osteogenic differentiation

Bone is a dynamic and metabolically active tissue that maintains a delicate balance between anabolic and catabolic processes throughout an individual’s life to preserve its structural integrity and strength. Bone remodeling is an ongoing process that occurs in response to mechanical stress, primarily involving bone resorption, facilitated by osteoclasts, and bone formation, mediated by osteoblasts. The dysfunction of such processes significantly contributes to various bone tissue diseases (Siddiqui and Partridge, 2016). BMSCs possess the remarkable ability for self-renewal and differentiation into various cell types. Notably, they can differentiate into osteoblasts, adipocytes, and chondrocytes (Pierce et al., 2019). Osteogenic/adipogenic differentiation disorder of BMSCs is the primary underlying cause of ONFH (Chen et al., 2020).

Recent studies have highlighted the impact of DNA methylation on osteogenic differentiation and its involvement in regulating bone metabolism (Malvicini et al., 2019; Li et al., 2019). Sun et al investigated the role of DNA methylation in steroid-induced osteonecrosis of femoral head (SONFH). Their results demonstrated that the proliferation ability of BMSCs in patients with SONFH was weakened. Additionally, the transcription level of the ABCB1 gene in BMSCs decreased, whereas its methylation modification level increased. The P-glycoprotein, encoded by this gene, played a crucial role in drug absorption and distribution. Notably, oral glucocorticoids served as the protein’s substrates. When BMSCs were treated with 5-aza, the expression of ABCB1 was rapidly restored (Sun et al., 2013). Further research by the same study group in the same patient population indicated that icariin-treated BMSCs reduced the methylation modification level of the CpG island of ABCB1, increased its gene expression, and promoted the osteogenic differentiation of BMSCs (Sun et al., 2015). Lysine-specific demethylase 5A (KDM5A) negatively regulates BMSC osteogenic differentiation by modulating H3K4me3 levels on the promoters of key osteogenic genes (Runx2, OCN, OPN) in SONFH (Yan et al., 2024). Human umbilical cord MSCs-derived exosomes (hucMSCs-exos) ameliorated alcohol-induced ONFH by inhibiting miR-25-3p DNA methylation and GREM1 expression in BMSCs (Tang et al., 2025). Dexamethasone trigger MC3T3E1 cells ferroptosis by promoting DNMT3a-mediated DNA methylation and downregulating Sirt1 expression in SONFH (Xiao et al., 2025).

DNA methylation has emerged as a potential therapeutic target for osteonecrosis of ONFH (Sun et al., 2015). A study on gene polymorphisms in the Chinese Han population with SONFN validated that polymorphisms in the ABCB1 and CYP450 genes were associated with the risk of steroid-induced ONFN. In particular, the CpG loci within the ABCB1 gene (ABCB1-1-192, ABCB1-2-32, and ABCB1-2-43) were identified with significantly different methylation levels (Huang et al., 2020). As a receptor within the Wnt signaling pathway, frizzled 1 (FZD1) is fundamental in osteoblast differentiation. In BMSCs from SONFH patients, hypermethylation of the FZD1 promoter CpG island reduced the expression of FZD1. Consequently, this inhibition affects the Wnt signaling pathway, weakens the proliferation ability of BMSCs, and hinders osteogenic differentiation, while also promoting adipogenic differentiation. Nonetheless, treatment with 5-aza can restore FZD1 expression by decreasing its methylation level, ultimately facilitating osteogenic differentiation and potentially serving as a therapeutic approach (Wu F. et al., 2019).

The inhibitory effect of the inflammatory factors such as TNF-α and IL-6, which are widely recognized as the main cytokines promoting apoptosis of bone cells or disturbance in bone metabolism associated with ONFH (Cheng et al., 2023; Ozawa et al., 2024), on osteogenic differentiation of BMSCs through epigenetic mechanisms remains a critical concern. The previous studies elucidated that the self-methylation of TNF-a and IL-6 plays a vital role in diseases such as obesity (Boonrong et al., 2024) and cognitive impairment (Rump and Adamzik, 2022). Boonrong et al. demonstrated that the hypomethylation in the TNF-α promoter correlated with elevated TNF-α levels and metabolic dysfunction in obese individuals, suggesting a feedforward loop between inflammation and epigenetic dysregulation. Furthermore, the hypermethylation of IL-6 regulated by DNMT1, contributed to the depression-like behaviors through inducing neuroinflammation in mice (Bai et al., 2023). An early study reported that TNF-α elevated the methylation level of the CpG region of Runx2, leading to the inhibition of the Wnt signaling pathway, subsequently hindering the differentiation of BMSCs into osteoblasts (Fang et al., 2019). However, the role of DNA methylation of TNF-α and IL-6 in ONFH is still unclear.

**FIGURE 2:**
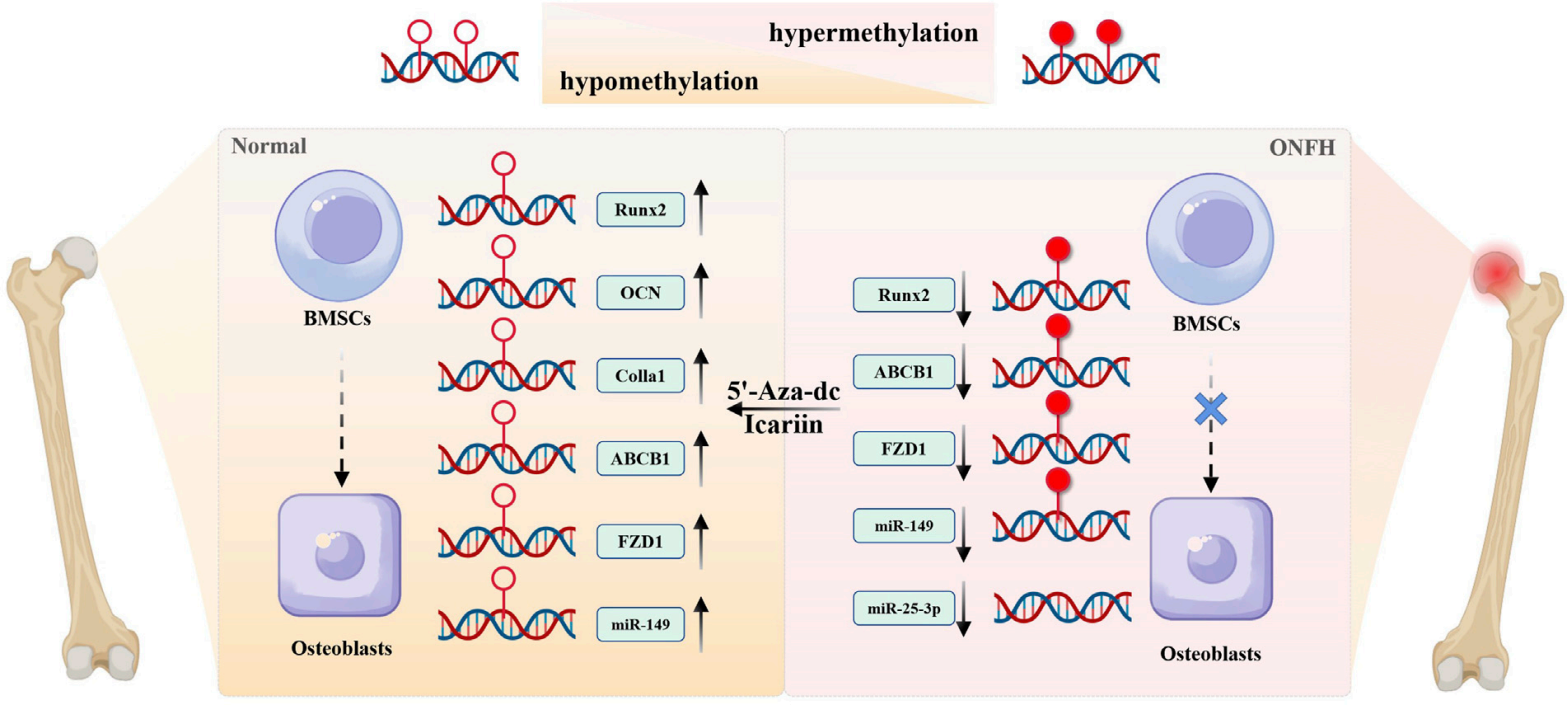
The role of DNA methylation on abnormal differentiation of BMSCs. Hypermethylation of osteogenic genes epigenetically silences their expression, thereby inhibiting osteogenic differentiation of BMSCs during the development of ONFH. DNA demethylating agents (e.g., 5-aza-dc) could reverse this hypermethylation, restoring expression level of genes such as Runx2, OCN, Colla1, ABCB1, FZD1, and promoting osteogenic differentiation from BMSCs to osteoblasts. Icariin exerts similar pro-osteogenic effects via DNA demethylation, highlighting its therapeutic potential for ONFH.

In conclusion, DNA methylation obstructs the BMSC-osteoblast differentiation by affecting the expression of osteogenic differentiation-related genes. Conversely, DNA demethylation reagents such as 5-aza-dc and icariin, can lower the methylation level of osteogenic genes, supporting osteogenic differentiation, and may potentially be developed as therapies (Figure 2).

### 3.2 The impact of DNA methylation on angiogenesis

Bone, a richly vascularized connective tissue, serves as the main source of oxygen, nutrients, hormones, neurotransmitters, and growth factors for bone cells. The vasculature is indispensable for appropriate bone development, regeneration, and remodeling (Peng et al., 2020). Blood vessels in bone develop through the process of angiogenesis. Their functional impairment can lead to various orthopedic diseases, including osteoporosis and osteonecrosis (Huang B. et al., 2016; Filipowska et al., 2017). In SONFH, angiogenesis may be involved in the repair of femoral head necrosis. Deferoxamine (DFO) can enhance angiogenesis and bone repair by upregulating the expression of key factors, including hypoxia-inducible factor-1α (HIF-1α), vascular endothelial growth factor (VEGF), bone morphogenetic protein-2 (BMP-2), and osteocalcin (OCN) (Li et al., 2015).

Xu et al. demonstrated that treating BMSCs the DNA methylation inhibitor 5-aza significantly increased the expression of endothelial markers, including CD31/PECAM1, CD105/ENG, and eNOS/VE-cadherin, thereby promoting angiogenesis (Xu R. et al., 2018). In their study using a tibial fracture model, Wang et al. observed that the expression of DNA methyltransferase Dnmt3b peaked on day 10 days after fracture and disappeared by day 28. Knocking out Dnmt3b led to delayed fracture repair, along with reduced numbers and volume blood vessels within the bone. Additionally, *in vitro* experiments showed that inhibiting Dnmt3b expression in human umbilical-vein endothelial cells resulted in reduced angiogenesis and inhibited endothelial cell migration. The mechanism of action is similar to that of CXCL12 and osteocalcin (osteopontin, OPN). The degree of methylation modification in the promoter region is associated with this process (Wang C. et al., 2018). These aforementioned results suggest that DNA methylation-mediated angiogenesis plays a substantial role in bone repair.

In their study, Yuan et al. conducted microRNA sequencing on the necrotic and normal parts of the femoral head. They found that miR-210 was significantly differentially expressed in necrotic tissue, with increased expression. Notably, when vascular endothelial cells were treated with demethylation reagents, the expression level of miR-210 further increased. Additionally, several angiogenesis-related genes (including VEGF, bFGF, TNF-α, and PCNA) were up-regulated, indicating that miR-210 facilitates angiogenesis and may have a potential therapeutic effect in SONFH (Yuan et al., 2016).

### 3.3 Theory of DNA methylation and apoptosis

Apoptosis refers to the programmed cell death regulated by genes. As scholars continue to investigate femoral head necrosis, the theory of apoptosis is gradually gaining recognition. Weinstein and their colleagues revealed a significant presence of osteocyte apoptosis in femoral head specimens from 14 SONFH patients (Weinstein et al., 2000). After extensive *in vitro* and *in vivo* experiments, the link between steroid- and alcohol-induced apoptosis of bone cells has been firmly established. Osteocyte apoptosis disrupts the sensory function of the cell mechanical network, leading to thinning and sparse trabecular bone. Ultimately, this process contributes to irreversible femoral head collapse. Nonetheless, inhibiting this mechanism can partially mitigate the progression of femoral head necrosis. The transcription factor p53 is vital in this context, activating both death receptor (FAS/TRAILR2)- and mitochondrial (BAX/BAK/BID/NOXA/PUMA/APAF1)-mediated apoptosis pathways by modulating the expression levels of binding site genes (Vousden and Lu, 2002). Abnormal hypermethylation in the promoter region of the p53 gene reduces the gene expression level, thereby affecting cell apoptosis. Moreover, apoptosis is also regulated by the impact of DNA methylation on the expression levels of Fas, Caspase-8, and p14ARF (Gopisetty et al., 2006).

TET3 is responsible for catalyzing the oxidation of 5-methylcytosine to 5-hydroxymethylcytosine (5hmc), substantially contributing to DNA demethylation (Wang et al., 2022; Zhang et al., 2023), which may play a crucial role in biological processes of ONFH by affecting gene expression (Wang et al., 2022). Early *in vivo* and *in vitro* studies demonstrated that the expression of TET3-5hmC increased in bone tissue associated with human steroid-induced ONFH and that the elevated TET3-5hmC levels correlated with weakened proliferation and increased apoptosis of osteocytes induced by steroid (Rath et al., 2024). These apoptosis-promoting effects of TET3-5hmC were medicated by PI3K/Akt signaling pathway. The knockdown on TET3 expression could counteract the inhibitory effect of steroid on the Akt pathway and suppress osteocyte apoptosis. On the other side, some studies have suggested that TET3 may promote repairing of spinal cord injury (Zhang et al., 2024) as shown by that TET3-mediated demethylation reshaped the methylation patterns of human umbilical cord mesenchymal stem cells (HUCMSCs), enabling their efficient one-step conversion into oligodendrocyte precursor cells (OPCs) for repairing spinal cord injury. Notably, this functional dichotomy may stem from tissue-specific gene regulatory network. These apoptosis-promoting and repair-promoting effects highlight the complexity in biological roles of TET3, suggesting that its therapeutic targeting might require precise spatiotemporal regulation. The TET3-5hmC-Akt axis represents a promising target of early intervention for steroid-associated ONFH.

### 3.4 DNA methylation and cartilage damage

In addition to subchondral osteonecrosis and collapse, the pathological process of femoral head necrosis also includes cartilage damage, degeneration and necrosis. In general, articular cartilage damage occurs during the early stages of ONFH and can lead to hip joint instability and femoral head collapse, further contributing to disease progression. Timely treatment of hip cartilage injuries can delay such progression and extend the window for surgical intervention (Xu et al., 2017; Zhao et al., 2017). Although chondrocytes do not undergo a remodeling process, DNA methylation remains vital in regulating chondrogenesis and maintenance, which is achieved by modulating the expression of chondrogenesis-related genes, including GDF5, SOX9, and MMP13 (Ramos and Meulenbelt, 2017). Moreover, during the differentiation of BMSCs into chondrocytes, the methylation level of the CpG site in the COL10A1 gene promoter region decreases. This demethylation process is associated with an increase in COL10A1 expression, ultimately promoting the differentiation of BMSCs into chondrocytes (Zimmermann et al., 2008).

A genome-wide DNA methylation sequencing study of hip articular cartilage in individuals with femoral head necrosis and femoral neck fracture revealed 480 hypermethylated sites and 1,335 hypomethylated sites associated with femoral head necrosis. Among these, the DNA methylation levels of several genes, namely, PDE4D, CARS, RUNX2, ADAMTS12, and LRP5, were decreased. Subsequent immunohistochemical experiments confirmed significant increased protein expressions of ADAMTS12, CARS, PDE4D, LRP5, and RUNX2 in the necrotic cartilage of the femoral head. Methylation yields a critical biological impact by influencing the methylation status of relevant genes in articular cartilage cells, hence regulating gene expression and exerting functional effects. Notably, cysteinyl-tRNA synthetase (CARS) has emerged as the most statistically distinct gene in DNA methylation sequencing results, encoding an aminoacyl-tRNA synthetase and is involved in regulating mitochondrial behaviors. Considering its potential in cartilage injury, further study is required to explore the role of CARS in ONFH-associated mitochondrial dysfunction (Wu J. et al., 2019). A recent study involving multiomics integration revealed 22 dysregulated genes in ONFH cartilage. Critically, MMP13 exhibited promoter hypomethylation coupled with transcriptional upregulation, driving extracellular matrix (ECM) degradation through collagen II cleavage. Conversely, GDF10 and CHI3L1 suppression was linked to promoter hypermethylation, leading to inflammatory changes and impairing BMP-mediated chondrogenesis. Dexamethasone induced DNA methylation changes, dysregulating effector molecules (MMP13, GDF10, and CHI3L1 et al.). This drives ECM degradation and impairs bone repair, culminating in femoral head collapse (Lu et al., 2024).

### 3.5 DNA methylation and signaling pathways

Genes involved in DNA methylation modifications are crucial in signaling pathways, affecting the abnormal differentiation of BMSCs in ONFH, maturation and differentiation of osteoclasts, and osteocyte apoptosis. The classical Wnt/β-catenin pathway significantly influences bone metabolism, generating dual effects: inhibiting the differentiation of BMSCs into chondrocytes and adipocytes, while promoting their differentiation into osteoblasts (Li et al., 2018; Hayashi and Nakashima, 2019).

The FZD protein, a 7-transmembrane receptor in the Wnt signaling pathway, interacts with Wnt ligands to activate downstream signaling. In particular, FZD1 has been implicated in promoting osteoblast differentiation and mineralization. Wu et al. they investigated DNA methylation in the promoter region of the FZD1 gene using BMSCs from patients with hormonal osteonecrosis of the femoral head. Notably, the FZD1 mRNA and protein levels were significantly reduced, inhibiting the Wnt/β-catenin signaling pathway and suppressing BMSC osteogenic differentiation, while also promoting adipogenic differentiation. However, treatment with the demethylation reagent 5-aza restored FZD1 expression, activating the Wnt/β-catenin pathway and ultimately enhancing BMSC osteogenic differentiation while inhibiting adipogenic differentiation (Wu F. et al., 2019). The osteoprotegerin (OPG)/receptor activator of nuclear factor kappa-Β ligand (RANKL)/RANK pathway remains essential in osteoclast maturation and differentiation. RANKL binds to RANK on the surface of osteoclasts, promoting their differentiation. OPG, secreted by osteoblasts, competes with RANKL, preventing RANKL from binding to RANK and inhibiting osteoclast function. Normally, OPG/RANKL/RANK is well-balanced. However, excessive alcohol and hormone intake can disrupt this balance, leading to osteoclast activation and reduced bone mass. Abnormal hypermethylation sites in the three genes have been observed in patients with hormonal and alcoholic femoral head osteonecrosis. The degree of OPG/RANKL/RANK hypermethylation may serve as a diagnostic marker for alcoholic femoral head osteonecrosis (Sun et al., 2021; Wang et al., 2021). The Akt signaling pathway has a pivotal impact on attenuating osteocyte proliferation through 3-5 hmC modification mediated by TET enzymes, eventually leading to apoptosis. Additionally, KEGG signaling analysis of osteocyte hMeDIP-5 hmC sequencing revealed that apart from Akt, the Notch and Wnt/β-catenin pathways are associated with hormone-induced DNA demethylation. In particular, Notch signaling promotes osteogenic differentiation, blood vessel formation, and bone formation (Ramasamy et al., 2014; Zhao et al., 2017).

## 4 DNA methylation-targeted therapy for ONFH: therapeutic potential and translational challenges

The reversible nature of DNA methylation, a key epigenetic mechanism, has provided innovative therapeutic strategies for diverse diseases such as cancer (Jamshidi et al., 2022; Lee et al., 2024), immune-related diseases (Gupta et al., 2023), and bone diseases (Grandi and Bhutani, 2020; de Nigris et al., 2021). DNMT inhibitors (DNMTis), particularly nucleoside analogs like 5-Azacytidine and 5-Aza-2′-deoxycytidine, exhibited clinical efficacy for types of tumors by restoring normal methylation patterns (Cheng et al., 2019). Moreover, numerous clinical trials investigating DNA methylation as an early diagnostic markers and therapeutic target for tumors, are either ongoing (NCT05764551, NCT03366116) or have been completed (NCT04568512). Indeed, the FDA have already approved blood tests based on DNA methylation biomarker technology to be applied in screening colorectal cancer.

Emerging evidence highlights DNA methylation as a pivotal regulator in ONFH, offering dual diagnostic and therapeutic opportunities. Genome-wide methylation analyses for bone tissue or blood from ONFH patients have identified disease-specific hypermethylation patterns in genes governing bone metabolism, including Runx2, OPG/RANKL/RANK signaling, CARS, and PDE4D (Wu J. et al., 2019; Sun et al., 2021; Wang et al., 2021). These sites might serve as new biomarkers for predicting the course and prognosis of ONFH. Thus, effective and specific DNA methylation inhibitors or activators could become a hotspot for new drug development to treat ONFH. Although studies on the clinical use of epigenetic drugs modulating aberrant DNA methylation pattern in early treatment of ONFH are at a very early stage, the development of epigenetic drugs targeting abnormal methylation of target genes still possess important application value.

## 5 Outlook

This paper reviews the distinct roles of DNA methylation and demethylation in ONFH. As the most common epigenetic modification, DNA methylation influences the progression of ONFH and the repair of the femoral head post-injury by regulating various factors, including the differentiation pathways of bone marrow stromal cells (BMSCs), angiogenesis-related factors, cell proliferation and apoptosis, chondrogenesis, and osteogenic/osteoclastic signaling pathways. On one hand, hypermethylation at CpG sites in genes such as ABCB1, FZD1, OCT4, miR-210, COL10A1, and OPG results in the downregulation of related proteins, inhibiting osteogenesis and accelerating the progression of ONFH (Table 1). Intervention with DNA methylation inhibitors could offer therapeutic benefits for ONFH. On the other hand, hypermethylation of genes like p53 and CARS can inhibit cell apoptosis and promote femoral head repair. As we know that the appropriate choice is considered for animal models, which should mimic the pathological mechanism of diseases, therefore, the rodent models (e.g., rats) are widely adopted due to their genetic homology with humans, similar physiologic and metabolic characteristics as humans, cost-effectiveness, and ease of breeding, in the studies on DNA methylation of ONFH. While, several critical limitations must be elucidated. Firstly, the faster metabolic rate of rodents accelerates glucocorticoid clearance as compared to humans, potentially underestimating the cumulative effects of chronic steroid exposure (Conzemius et al., 2002; Xu J. et al., 2018). To address this concern, adjusted doses (e.g., 20–40 mg/kg methylprednisolone intramuscularly in divided doses over 3 days) are recommended to reflect human-like pathological progression (Tao et al., 2017; Li et al., 2023). Secondly, rodents exhibit enhanced capacity in bone repairing, which may mask progression of ONFH. To avoid this problem, the extended observation periods (≥4 weeks) are necessary to evaluate stable necrosis and femoral head collapse (Fan et al., 2011; Huang S. L. et al., 2016; Tao et al., 2017). Finally, standardized evaluation criteria are essential in ONFH studies, including but not limited to, histological staining (H&E, Masson) and imaging methods (MRI T1-weighted sequences, micro-CT), which are suggested to be used to quantify necrosis severity (Qin et al., 2006; Yang et al., 2009)”.

**TABLE 1:** Summary of the genes involved in DNA methylation regulation in ONFH.

Gene	DNA methylation	Biological function	Signaling pathway	References
ABCB1	hypermethylation	aberrant osteogenic differentiation	—	Reddy and Natarajan, (2015), Koch et al. (2018), Agha et al. (2019)
miR-210	hypomethylation	angiogenesis	—	Greenberg and Bourc’his (2019)
FZD1	hypermethylation	abnormal osteogenic/adipogenic differentiation	Wnt/β-catenin	Van Meurs et al. (2019)
TET3	hypomethylation	cell apoptosis and weakened proliferation	PI3K/AKT	Edwards et al. (2017)
ADAMTS12	hypomethylation	cartilage damage	—	Zhou, G.S. (2009)
CARS	hypomethylation	cartilage damage	—	Zhou, G.S. (2009)
OPG/RANKL	hypermethylation	osteoclast maturation and differentiation	OPG/RANKL/RANK	Sun et al. (2013), Sun et al. (2015)
MMP13	hypomethylation	cartilage damage	—	Lu et al. (2024)
CHI3L1	hypermethylation	cartilage damage	—	Lu et al. (2024)
GDF10	hypermethylation	cartilage damage	—	Lu et al. (2024)
miR-25-3p	hypomethylation	osteogenic differentiation	miR-25-3P/GREM1	Tang et al. (2025)
KDM5A	hypermethylation	aberrant osteogenic differentiation	—	Yan et al. (2024)

Although current research has not completely elucidated the mechanisms by which DNA methylation affects ONFH, the significance of DNA methylation in the diagnosis and treatment of ONFH is substantial. Scientific research should not be limited to animal and *in vitro* experiments; studies in real-world settings should also be emphasized. Continued research will enhance the understanding of DNA methylation’s role in ONFH, providing new theoretical foundations, diagnostic approaches, and therapeutic strategies, and offering insights for the development of new drugs for treating ONFH.
